# Cardiovascular Pharmacogenomics: Current Status and Future Directions—Report of a National Heart, Lung, and Blood Institute Working Group

**DOI:** 10.1161/JAHA.111.000554

**Published:** 2012-04-24

**Authors:** Kiran Musunuru, Dan M. Roden, Robin Boineau, Michael R. Bristow, Timothy A. McCaffrey, Christopher Newton-Cheh, Dina N. Paltoo, Yves Rosenberg, Jay G. Wohlgemuth, Issam Zineh, Ahmed A. K. Hasan

**Affiliations:** Brigham and Women's Hospital, Boston and Harvard University, Cambridge, MA (K.M.); Vanderbilt University Medical Center, Nashville, TN (D.M.R.); National Heart, Lung, and Blood Institute (NHLBI), Bethesda, MD (R.B., D.N.P., Y.R., A.A.K.H.); University of Colorado Cardiovascular Institute, Aurora, CO (M.R.B.); George Washington University Medical Center, Washington, DC (T.A.M.); Massachusetts General Hospital, Harvard Medical School, Boston, MA (C.N.-C.); Quest Diagnostics Nichols Institute, San Juan Capistrano, CA (J.G.W.); U.S. Food and Drug Administration, Silver Spring, MD (I.Z.)

**Keywords:** genetic polymorphisms, genetics, pharmacogenetics, pharmacogenetics anticoagulants, pharmacogenetics cholesterol

## Introduction

The National Heart, Lung, and Blood Institute (NHLBI) convened a Working Group on January 7, 2011, at George Washington University in Washington, DC, to provide recommendations to the NHLBI that would guide informed decisions on research directions and priorities in the field of cardiovascular pharmacogenomics. This meeting was timed to follow the *New Frontiers in Personalized Medicine: Cardiovascular Research and Clinical Care* conference held the previous day and cosponsored by the NHLBI, Personalized Medicine Coalition, American College of Cardiology, American Medical Association, and Cheney Cardiovascular Institute at George Washington University. The conference brought together leaders from academia, industry, and government to (1) discuss personalized medicine's current and potential impact on cardiovascular patient outcomes; (2) review emerging technologies and applications that may shape the field in the future; (3) discuss the results of an American College of Cardiology survey examining personalized medicine adoption rates among US cardiologists; (4) identify the barriers to adoption of pharmacogenetics-based personalized medicine in cardiovascular practice; and (5) develop recommendations for next steps with an emphasis on actions and evidence generation that are needed for adoption and improved quality of cardiovascular patient care.

The goals of the NHLBI Working Group were to review the discussion and recommendations from the *New Frontiers in Personalized Medicine: Cardiovascular Research and Clinical Care* conference; to identify areas and challenges that should be addressed to further the field of cardiovascular pharmacogenomics and increase its adoption into the clinical setting; to develop recommendations and priorities for implementing current pharmacogenomics-based treatment modalities; and to identify future research needs related to cardiovascular pharmacogenomics.

## Conference Summary

Cardiovascular disease remains the number one cause of death in the United States and will likely soon become the number one cause of death globally. At the same time, new understandings of individual characteristics including genetic variations are increasingly reshaping our ability to treat various aspects of cardiovascular disorders. The *New Frontiers in Personalized Medicine: Cardiovascular Research and Clinical Care* conference brought together leaders from academia, industry, and government to address the critical issues facing the future of cardiovascular research and clinical care in science, business, and policy.

Four major goals for the next 5 to 10 years were outlined as follows: (1) to establish standards of quality for the research enterprise, (2) to establish robust systems for more rapid evidence generation, (3) to harmonize regulatory and reimbursement standards, and (4) to develop innovative partnerships to accelerate the development and implementation of personalized medicine applications. Three examples of personalized medicine that are primed for widespread clinical use were highlighted as follows: (1) the use of pharmacogenomic algorithms to guide the dosing of warfarin therapy; (2) the use of blood cell gene expression profiles to detect or rule out cardiac transplant rejection; and (3) the targeted sequencing of areas of genes (resequencing) implicated in long-QT syndromes to identify causal mutations in affected individuals and to screen family members for the same mutations.

Several new technologies with the potential to result in new tests and new drugs were discussed, including deep sequencing of DNA and RNA, biomarker panels, polymerase chain reaction technology, proteomics, and metabolomics. In addition, strategies to bridge the much-cited “valley of death” between basic science discoveries and development of successful drugs included the use of genomics data to establish the relevance of drug targets in humans before starting clinical trials, the use of intermediate biomarker or imaging endpoints to make the decision to proceed with or terminate drug candidates early in the development process, and early identification of subpopulations most likely to benefit from therapy, for example, by using common genetic polymorphisms (individually or combined into scores) or gene expression profiles.

It was noted that few cardiologists report feeling knowledgeable with respect to personalized medicine, highlighting the particular need for education aimed not only at patients but also at providers if widespread adoption of a test is to be achieved. A number of potential solutions to overcoming clinical and regulatory barriers to personalized cardiovascular care were addressed, including the education of providers in principles of basic science that are relevant to personalized medicine; integrating personalized medicine practices into the clinical workflow using electronic platforms; “real-world” studies to assess whether personalized medicine improves outcomes when used directly in a clinical practice context; and incentives for providers and patients to improve adherence to personalized medicine practices.

## Challenges for Cardiovascular Pharmacogenomics

Guided by the proceedings of the *New Frontiers in Personalized Medicine: Cardiovascular Research and Clinical Care* conference, the NHLBI Working Group characterized and discussed challenges for cardiovascular pharmacogenomics in 5 domains as follows: clinical needs, clinical validation, information delivery, education and compliance, and cost-effectiveness. For the purpose of discussion, the Working Group adopted the International Conference on Harmonization E15 harmonized definition of pharmacogenomics as “the study of variations of DNA and RNA characteristics as related to drug response.” The majority of the subsequent discussion focused on the study of DNA sequence variation as related to drug response.

### Clinical Needs

The Working Group sought to identify and prioritize the most pressing clinical needs to focus research and translational efforts. In particular, 3 areas of emerging pharmacogenomic applications were reviewed: anticoagulation (warfarin), antiplatelet therapy (clopidogrel), and lipid-lowering therapy (statins).

### Warfarin

Warfarin, used widely for the prevention and treatment of thromboembolic disease, is challenging to use because of highly variable responses among patients and even within an individual patient. Patients receiving warfarin anticoagulation require frequent monitoring of blood clotting activity as measured by the prothrombin time (international normalized ratio) particularly in the immediate period after the initiation of warfarin therapy—with significant risk of either thromboembolism if the warfarin dose is too low or bleeding if the dose is too high. Polymorphisms in 2 genes, *CYP2C9* (cytochrome P450 2C9) and *VKORC1* (vitamin K epoxide reductase complex subunit 1), account for more than one third of the interindividual variation in stable therapeutic dosing of warfarin.^1–5^

An early small clinical trial evaluated an algorithm that incorporated the *CYP2C9* and *VKORC1* polymorphisms in an attempt to better predict the optimal starting warfarin dose.^6^ When compared with the usual practice (choosing a starting dose using clinical judgment), the pharmacogenomic algorithm did not improve the safety of warfarin initiation—the number of out-of-range international normalized ratios during the initiation period was unchanged—although it did reduce the dosing changes needed to achieve stable anticoagulation.^6^ Subsequent small prospective clinical trials suggested that addition of genetic information could improve the safety and efficacy of warfarin use, particularly in guiding the choice of maintenance dosing.^7–9^

A large warfarin pharmacogenomics study, the Medco-Mayo Warfarin Effectiveness Study with almost 4000 individuals, was designed to test whether the use of genotype information could reduce the incidence of hospitalizations from warfarin-related adverse effects.^10^ It was performed in a community practice setting rather than as a randomized prospective study. In this “real-world” study setting, *CYP2C9* and *VKORC1* genotypes were determined in about 900 patients in 23 prescription benefit plans who were starting warfarin therapy, the genotype data were made available to their providers (with no subsequent communication with the providers), and outcomes were compared with those in 2700 historical controls in the same 23 plans for whom genotypes had not been determined. Two external control groups from a different set of 56 plans, one concurrent with the genotyped group and the other concurrent with the historical control group, were also followed. Within a 6-month follow-up period, there was a 31% reduction of hospitalization in the genotyped patients compared with the controls, with a 28% reduction of hospitalization due to bleeding or thromboembolism; there were no significant differences between the external control groups.^10^

The study design of Medco-Mayo Warfarin Effectiveness Study has been criticized for using historical controls instead of contemporaneous controls, and for the relatively slow turnaround time of *CYP2C9* and *VKORC1* genotyping, with providers receiving this data several weeks after initiation of warfarin. Of note, the delay in obtaining the genotyping information indicates that the benefits of the data came in guiding maintenance dosing, rather than the initial dosing of warfarin. Studies of alternative designs are underway, such as the Clarification of Optimal Anticoagulation Through Genetics study, an NHLBI-sponsored prospective randomized clinical trial that will enroll up to 1200 participants and compare a clinical algorithm for determining the dosing for warfarin initiation to a pharmacogenomic algorithm using *CYP2C9* and *VKORC1* genotypes.^11^

The results from Medco-Mayo Warfarin Effectiveness Study and other warfarin trials suggest that anticoagulation has the potential to be an early widely adopted pharmacogenomic application in cardiovascular medicine. Of note, the US Food and Drug Administration—approved label for warfarin has been revised to include specific dosing guidelines for patients with known *CYP2C9* and *VKORC1* genotypes. The availability of alternatives to warfarin, such as the newly approved dabigatran, also makes possible a therapeutic strategy whereby a patient with *CYP2C9* and *VKORC1* genotypes and other clinical characteristics that presage higher risk for warfarin-related adverse effects might be prescribed a different anticoagulant, assuming the patient does not have contraindications to that anticoagulant (in the case of dabigatran, severe renal impairment or high risk of bleeding).

### Clopidogrel

Although dual antiplatelet therapy with aspirin and clopidogrel is now standard therapy for acute coronary syndrome patients, particularly those undergoing percutaneous coronary intervention (PCI),^12,13^ it is clear that patients display variable responses to clopidogrel therapy. A major contributor to this variability is the common *2 loss-of-function variant in *CYP2C19*, encoding a hepatic cytochrome P-450 2C19 enzyme important for clopidogrel bioactivation.^14–16^ Three large studies of mostly post—acute coronary syndrome and/or post-PCI patients on clopidogrel therapy (TRITON-TIMI 38 [Trial to Assess Improvement in Therapeutic Outcomes by Optimizing Platelet Inhibition with Prasugrel Thrombolysis In Myocardial Infarction 38], FAST-MI [French Registry of Acute ST-Elevation and Non-ST-Elevation Myocardial Infarction], and AFIJI [Appraisal of Risk Factors in Young Ischemic Patients Justifying Aggressive Intervention]) identified at least one copy of CYP2C19*2 in ≈30% of individuals. In all 3 studies, reduced-function allele carriers experienced significantly higher rates of cardiovascular death, myocardial infarction, and stroke.^17–19^ Considering the totality of evidence, the US Food and Drug Administration—approved clopidogrel label was updated to include a “boxed warning” to underscore that individuals carrying 2 reduced-function *CYP2C19* alleles—so-called “poor metabolizers”—experience diminished effectiveness of the drug at standard dosing and that alternative therapeutic strategies should be considered in these patients.

Subsequent studies have begun to clarify when clopidogrel pharmacogenomics may be helpful in guiding therapy. A meta-analysis of 9 clopidogrel studies with a combined 10 000 participants—comprising mostly PCI patients, with over half being treated for an acute coronary syndrome—found that carriers of reduced-function *CYP2C19* alleles suffered a 57% increase in risk of cardiovascular death, myocardial infarction, or ischemic stroke compared with noncarriers.^20^ The increased risk affected carriers of 2 reduced-function *CYP2C19* alleles (76% increase) as well as carriers of 1 reduced-function *CYP2C19* allele (55% increase). Notably, there was an almost tripling of risk of stent thrombosis in reduced-function *CYP2C19* allele carriers.

In contrast, genotype data from participants in the CURE (Clopidogrel in Unstable Angina to Prevent Recurrent Events) and ACTIVE A (Atrial Fibrillation Clopidogrel Trial with Irbesartan for Prevention of Vascular Events A) trials indicated that the relative cardiovascular risk reduction seen with clopidogrel treatment (vs placebo) was similar for carriers of reduced-function *CYP2C19* alleles and noncarriers.^21^ However, very few of the patients in either trial underwent PCI with stent placement (14.5% in CURE). For participants in the PLATO (Platelet Inhibition and Patient Outcomes) trial, in which 64% underwent PCI with stent placement, reduced-function *CYP2C19* allele carriers experienced a higher event rate on clopidogrel than noncarriers within 30 days of initiation of therapy, but in the long term there was no difference in the event rate on clopidogrel.^22^ Together, these studies suggest that (1) the effect of reduced-function *CYP2C19* alleles may be more relevant in the acute setting rather than the long term, and (2) *CYP2C19* genotyping may be more useful in higher-risk patients (ie, those undergoing PCI with stent placement in the setting of an acute coronary syndrome) than in lower-risk patients.

Unlike with warfarin, there have not yet been any trials assessing whether clinical decision-making informed by knowledge of *CYP2C19* genotypes improves clinical outcomes. Therapeutic strategies that await testing include (1) giving carriers of reduced-function *CYP2C19* alleles a higher-than-standard dose of clopidogrel or (2) giving carriers alternative thienopyridines such as prasugrel and ticagrelor. Several hospitals have already begun planning for on-site, point-of-care *CYP2C19* genotype testing of PCI patients in anticipation of implementing one of these strategies. The relative merits of focused point-of-care testing of acute patients versus routine pretesting for *CYP2C19* genotype (as well as other pharmacogenomic data) in all at-risk patients in the outpatient setting (eg, cardiology clinic) remain to be determined.

### Statins

Statins are among the most widely prescribed drugs in the world, used for the reduction of plasma low-density lipoprotein cholesterol levels and the prevention of cardiovascular disease. Development of pharmacogenomic tests that could predict response to statin therapy might therefore have significant clinical implications. The Trp719Arg (W719R) variant of the *KIF6* (kinesin-like family 6) gene (also designated rs20455) was identified in a small case-control study of myocardial infarction.^23^ Follow-up studies suggested that the variant might have value for a pharmacogenomic application. In a subset of the WOSCOPS (West of Scotland Coronary Prevention Study) trial, carriers of the *KIF6* variant experienced greater protection against coronary heart disease with pravastatin therapy compared with noncarriers.^24^ In the CARE (Cholesterol and Recurrent Events) trial, there was a smaller difference in response to pravastatin therapy between *KIF6* variant carriers and noncarriers, with the trend favoring the carriers.^24^ A similar trend favoring carriers was observed in the PROSPER (Prospective Study of Pravastatin in the Elderly at Risk) trial in subjects with a prior history of vascular disease.^25^ Finally, in the PROVE IT-TIMI 22 (Pravastatin or Atorvastatin Evaluation and Infection Therapy: Thrombolysis in Myocardial Infarction 22) study, *KIF6* variant carriers obtained significantly greater benefit from intensive statin therapy (atorvastatin 80 mg daily) compared with moderate statin therapy (pravastatin 40 mg daily) than did noncarriers.^26^

Published subsequent to the meeting of the Working Group, an analysis of the HPS (Heart Protection Study) clinical trial in subjects with a history of coronary or peripheral vascular disease found no difference between *KIF6* variant carriers and noncarriers in their response to therapy (simvastatin vs placebo), with both groups receiving significant benefit from statin therapy.^27^ A similar observation was made in a study from the JUPITER (Justification for the Use of Statins in Primary Prevention: An Intervention Trial Evaluating Rosuvastatin) trial, where *KIF6* W719R variant noncarriers experienced as much protection from statin therapy (rosuvastatin vs placebo) as did carriers.^28^ As has been observed for clopidogrel use in reduced-function *CYP2C19* allele carriers (as discussed previously), differences in study designs, study endpoints, risk characteristics of the study populations, and the statin drugs and dosages used in the various trials may account for the conflicting findings with the *KIF6* W719R variant. Another possibility is that the later, larger studies signify that the original findings with the *KIF6* W719R variant were simply the play of chance.

A single variant in *SLCO1B1*, which encodes a hepatic uptake transporter, has been associated with a 17-fold increased risk of myopathy with high-dose (80 mg/d) simvastatin (see later); the finding was replicated albeit with lower odds ratios at a dose of 40 mg/d.^29^ Thus, the *SLCO1B1* variant could potentially be used to identify susceptible individuals in whom to avoid the use of high-dose simvastatin; the recent US Food and Drug Administration relabeling of the drug (to avoid the 80-mg/d dose altogether) should also reduce population risk.

### Development of Further Applications

Besides the 3 areas addressed previously—anticoagulation, antiplatelet therapy, and lipid-lowering therapy—the Working Group noted additional areas in cardiovascular medicine for which pharmacogenomics could potentially be of significant clinical impact, including treatment of hypertension, treatment of heart failure, and prediction of the development of cardiomyopathy with chemotherapeutic drugs.

The Working Group also recognized that recent advances in human genetics have made it possible to identify many DNA variants with potential pharmacogenomic relevance. During the past few years, genome-wide association studies (GWASs) have been enormously successful in identifying variants associated with cardiovascular phenotypes, including blood lipid levels,^30,31^ blood pressure,^32,33^ and coronary artery disease.^34,35^ GWASs have also been successfully been performed on medication responses, for example, the finding of the statin myopathy—associated *SLCO1B1* variant described earlier.^29^ A GWAS on the response of platelet aggregation to clopidogrel therapy identified variants near the *CYP2C19* gene,^36^ the same gene known to harbor reduced-function alleles that affect clinical responses to the drug, as described previously; conditioning the analysis on the CYP2C19*2 allele eliminated the GWAS signal, indicating that this variant drives the result. In both of these cases, the GWASs pointed to genes already functionally linked to the drugs of interest. It can be anticipated that future GWASs on medication responses will implicate novel genes with unclear function, understanding of which might ultimately lead to new pharmacogenomic applications and therapeutic strategies. Accordingly, investment in GWASs and, in particular, follow-up studies on GWAS discoveries—identifying causative genes, DNA variants, and biological mechanisms using cellular and organismal model systems, systems biology approaches, integration with bioinformatics databases, targeted candidate gene resequencing, and other methodologies—is warranted.

### Clinical Validation

The Working Group noted that the gold standard for pharmacogenomic applications (as with all clinical interventions) is a prospective trial with treatment determined by genotype and with a clinical endpoint as the primary outcome, but it also recognized that it would be very difficult to sustain and fund a large number of such trials, and that in some cases the weight of evidence from observational studies may be so compelling as to render prospective trials unnecessary. Thus, there is a need to carefully choose which pharmacogenomic applications warrant funding of definitive prospective randomized trials. This would be best served by convening expert panel meetings or conferences to periodically evaluate the latest evidence for several cardiovascular pharmacogenomic clinical applications and recommend whether to provide funding for large clinical trials. The Working Group also acknowledged that large trials will not always be practical or optimal to test many potential pharmacogenomic applications, highlighting the need for innovative clinical trial designs to test pharmacogenomic strategies. One such example is provided by the Medco-Mayo Warfarin Effectiveness Study warfarin trial, as described previously. Another example is to compare outcomes between community practice groups cluster randomized to pharmacogenomic strategies and groups that maintain the usual practices. To promote innovation in clinical trial design, the Working Group envisions expert panel meetings or conferences with the express purpose of devising clinical trial designs specifically suited to the testing of pharmacogenomic strategies.

The examples of the reduced-function *CYP2C19* and *KIF6* W719R variants, as described previously, highlight the need for a substantial evidence base comprising numerous studies in order to define whether a DNA variant truly predicts a clinical outcome and, if so, which patient populations are most likely to benefit from a proposed pharmacogenomic application using the variant. Recognizing the need for validation studies for many future pharmacogenomic applications, the Working Group discussed means by which to promote the inclusion and utilization of DNA collections in as many clinical trials and clinical populations—that is, large cohorts with close surveillance—as possible.

One mechanism would be for funding agencies to underwrite the costs of storage of sample collections from clinical trials as well as large healthcare systems (eg, one of the US Veterans Affairs regional healthcare systems) in which electronic record-keeping and close clinical follow-up occur, which would empower future large-scale pharmacogenomic observational studies. Another mechanism would entail funding agencies requiring DNA banking as a condition for future clinical studies to receive funding; an alternative would be for proposed clinical studies that include DNA banking to receive priority over clinical studies that do not. A third mechanism would be for funding agencies to provide extra funding for studies using DNA collections through existing ancillary studies programs. Finally, it could be helpful to convene an expert panel meeting or conference in which participants would seek to achieve consensus on the optimal use of DNA collections in pharmacogenomics research.

Finally, the Working Group recognized that, in the interest of developing a pipeline of new pharmacogenomic applications, funding would be needed for small-to-medium size proof-of-concept clinical studies, numbering several dozen to several hundred participants, to validate preliminary pharmacogenomics findings from basic science studies or post-hoc analyses of clinical trials. This would help to screen out false-positive findings before much effort and resources are devoted to larger clinical studies.

### Information Delivery

The Working Group identified barriers to implementation, such as the need for fast delivery of pharmacogenomic data in the point-of-care setting in order to enable clinical decisions, the reliance on clinical workflow rules to streamline a busy clinical schedule, and lack of utilization even when providers are aware that pharmacogenomics may be helpful. Pharmacogenomic guidelines, reference databases, and electronic platforms to integrate personal pharmacogenomic data into the clinical workflow would all be helpful in overcoming these barriers. Innovative strategies are needed to promote the widespread adoption of pharmacogenomic clinical applications by cardiovascular providers.

One such strategy would be the use of information technology to integrate pharmacogenomics into the clinical workflow, which would follow a “learning by doing” model. For example, a provider using a computerized provider order entry system to prescribe clopidogrel for a patient would be asked whether the patient is known to have reduced-function *CYP2C19* alleles and, if not, would be prompted to consider ordering a genotyping test. Extending this model, if the computerized provider order entry were linked to the patient's medical record and found that the patient was known to have reduced-function *CYP2C19* alleles, the system would prompt the provider to consider using an alternative antiplatelet medication. Thus, preemptive genotyping—placing data in computerized provider order entry—enabled electronic medical records—is another strategy that needs further evaluation.

More generally, information delivery would be facilitated by the development of a publicly available database, modeled on the highly regarded Online Mendelian Inheritance in Man database (http://www.ncbi.nlm.nih.gov/omim), that would curate functional and pharmacogenomic data as well as evaluate the evidence related to specific DNA variants. The Pharmacogenomics Knowledge Base (PharmGKB; http://www.pharmgkb.org/), initially founded as part of the National Institutes of Health's Pharmacogenomics Research Network, includes as a mission to serve as a central repository from which pharmacogenomic guidelines, reference databases, and electronic platforms could all draw. The Pharmacogenomics Research Network and PharmGKB have also organized the Clinical Pharmacogenomics Implementation Consortium^37^ to provide specific evaluations of the strength of evidence for specific drug-gene variant relations that might support their implementation in practice; this represents another critical need in the field.

### Education and Compliance

The Working Group recognized that many of the same barriers and potential solutions for information delivery are relevant to education and compliance, and that education must be aimed not only at patients but also at providers. One means by which to widely disseminate recommendations on pharmacogenomic applications would be to incorporate them into clinical guidelines, as in the Clinical Pharmacogenomics Implementation Consortium effort. This could be achieved by including cardiovascular pharmacogenomics experts on high-profile guidelines committees such as the American College of Cardiology/American Heart Association Task Force on Practice Guidelines, the National Cholesterol Education Program, and the Joint National Committee on Prevention, Detection, Evaluation, and Treatment of High Blood Pressure. Given the current push to encourage compliance with clinical guidelines through a variety of incentive programs (eg, Medicare pay-for-performance initiatives), it is to be expected that inclusion of pharmacogenomics recommendations into clinical guidelines would, in time, result in broad adoption of pharmacogenomic practices.

Another priority should be the establishment of training programs in pharmacogenomics—incorporating formal training in clinical pharmacology, genetics, biostatistics, informatics, epidemiology, and clinical trial design—that could be supported by mechanisms such as the National Institutes of Health T32 type grants. This would serve 2 objectives: to promote pharmacogenomics research by developing investigators with the optimal set of skills needed to perform the research, and to create a cohort of experts widely distributed across many institutions who could serve as local leaders to educate others in the use of pharmacogenomic applications.

Finally, efforts should be made to promote the training of established clinicians as well as clinical trainees in the principles of genetics and pharmacogenomics—the former through continuing medical education activities, the latter through curricula introduced in residency and fellowship programs.

### Cost-Effectiveness

The Working Group recognized that pharmacogenomic testing would add costs that are easy to assess but would also yield cost savings, clinical benefits, and other types of “value” that are harder to quantify. The Working Group noted that there are no established models to use in this relatively new field, requiring the recruitment of expertise to develop such models. This process could be initiated by convening expert panel meetings or conferences aimed at achieving consensus on the “best practices” for the use of biostatistics and cost-effectiveness analyses in pharmacogenomic studies. These models will be essential to persuade various stakeholders to support the implementation of specific pharmacogenomic applications and to ensure that resources and efforts in clinical development of pharmacogenetic tests (translational studies and clinical validation) are appropriately allocated.

One analysis of *CYP2C9* and *VKORC1* genotype-guided dosing of warfarin found that, with a cost of $400 and a 3-day delay for genotyping, the marginal cost-effectiveness would be $170 000 per quality-adjusted life-year; with a cost of $200 and a 24-hour turnaround time, the marginal cost-effectiveness would fall to $50 000 per quality-adjusted life-year, and genotyping would be cost-saving if the cost fell below $40 per test.^8^ Thus, with cost-effectiveness improving as the costs associated with pharmacogenomic tests fall, it will be critical to develop inexpensive, rapid, and extremely accurate genotyping and targeted resequencing assays for a variety of clinical applications. To date, the dramatic decrease in genotyping and sequencing costs over the past 15 years—such that the much-heralded “$1000 genome” is likely to become reality within a few years—has largely been driven by high-volume research applications (eg, GWASs of hundreds of thousands of individuals, and now exome and whole-genome sequencing studies in thousands of individuals) funded by public agencies like the National Institutes of Health and charitable foundations like the Wellcome Trust. It can be expected that similar sources of funding will be needed to stimulate the development of technology for low-cost pharmacogenomic tests that are appropriate for widespread, routine clinical use or for preemptive testing coupled to sophisticated electronic medical record systems.

## Recommendations for Scientific Investment

The Working Group identified a set of research and policy priorities designed to facilitate the development and adoption of cardiovascular pharmacogenomics in patient care. High-priority research programs and infrastructure needs identified include the following:

1. Research projects targeted to high-need areas, including the following:
  - Small-to-medium size proof-of-concept clinical studies (ie, several dozen to several hundred participants) to validate preliminary pharmacogenomics findings from basic science studies or post-hoc analyses of clinical trials.
  - Studies to reap the benefit of GWAS locus discovery by identifying causative genes, DNA variants, and biological mechanisms using cellular and organismal model systems, systems biology approaches, integration with bioinformatics databases, targeted candidate gene resequencing, and other methodologies.
  - The development of inexpensive, rapid, and extremely accurate genotyping and targeted resequencing assays for clinical use, acknowledging that current methods are geared toward research applications rather than clinical use.
2. Training programs for young investigators as well as established investigators to develop skills in cardiovascular pharmacogenomics. Such programs could include formal training in clinical pharmacology, genetics, biostatistics, informatics, epidemiology, clinical trial design, and health outcomes and cost-effectiveness research.
3. Promotion of the inclusion and utilization of DNA collections in clinical trials and for clinical populations for pharmacogenomics analyses by:
  - Providing funding for storage of sample collections.
  - Requiring or incentivizing DNA banking for funded clinical studies.
  - Providing funding for studies using DNA collections via the ancillary studies program.
4. Expert panel meetings or conferences to:
  - Periodically evaluate the latest evidence for several cardiovascular pharmacogenomic clinical applications and recommend whether to provide funding for large clinical trials. An example of a clinical application that is a high priority for evaluation is the use of *CYP2C19* genotypes to guide clopidogrel therapy.
  - Achieve consensus on the optimal use of DNA collections in pharmacogenomics research.
  - Propose innovative clinical trial designs to test pharmacogenomic strategies, acknowledging that large prospective randomized clinical trials will not always be practical or optimal to test many potential pharmacogenomic applications. An example is to compare outcomes between community practice groups cluster randomized to pharmacogenomic strategies and groups that maintain the usual practices.
  - Achieve consensus on the “best practices” for the use of biostatistics and cost-effectiveness analyses in pharmacogenomic studies.
  - Propose innovative strategies to promote the widespread adoption of pharmacogenomic clinical applications by cardiovascular providers. An example is the use of information technology to educate providers and to integrate pharmacogenomics into the clinical workflow.
5. Inclusion of cardiovascular pharmacogenomics experts on guidelines committees to facilitate the inclusion of pharmacogenomics recommendations in clinical guidelines. Such committees include the following:
  - American College of Cardiology/American Heart Association Task Force on Practice Guidelines (eg, clopidogrel pharmacogenomics).
  - National Cholesterol Education Program (eg, statin pharmacogenomics).
  - Joint National Committee on Prevention, Detection, Evaluation, and Treatment of High Blood Pressure (eg, beta-blocker pharmacogenomics).
6. Engagement with the US Food and Drug Administration and other regulatory agencies to develop guidance documents on the types of evidence required to gain approval of diagnostic biomarkers and pharmacogenomically targeted therapies.
